# Behavior of Oxidative Stress Markers in Alcoholic Liver Cirrhosis Patients

**DOI:** 10.1155/2016/9370565

**Published:** 2016-12-15

**Authors:** Marina Galicia-Moreno, Dorothy Rosique-Oramas, Zaira Medina-Avila, Tania Álvarez-Torres, Dalia Falcón, Fátima Higuera-de la tijera, Yadira L. Béjar, Paula Cordero-Pérez, Linda Muñoz-Espinosa, José Luis Pérez-Hernández, David Kershenobich, Gabriela Gutierrez-Reyes

**Affiliations:** ^1^HIPAM Lab, Experimental Medicine Unit, School of Medicine, Universidad Nacional Autónoma de México, Hospital General de México, Mexico City, Mexico; ^2^Departamento de Farmacología, Facultad de Medicina Mexicali, UABC, Mexicali, BC, Mexico; ^3^Liver Clinic, Gastroenterology Service, Hospital General de México, Mexico City, Mexico; ^4^Blood Bank Service, Hospital General de México, Mexico City, Mexico; ^5^Liver Unit and Molecular Medicine, University Hospital, Universidad Autónoma de Nuevo León, Monterrey, NL, Mexico; ^6^Instituto Nacional de Ciencias Médicas y Nutrición Salvador Zubirán, Mexico City, Mexico

## Abstract

Alcohol is the most socially accepted addictive substance worldwide, and its metabolism is related with oxidative stress generation. The aim of this work was to evaluate the role of oxidative stress in alcoholic liver cirrhosis (ALC). This study included 187 patients divided into two groups: ALC, classified according to Child-Pugh score, and a control group. We determined the levels of reduced and oxidized glutathione (GSH and GSSG) and the GSH/GSSG ratio by an enzymatic method in blood. Also, protein carbonyl and malondialdehyde (MDA) content were estimated in serum. MDA levels increased in proportion to the severity of damage, whereas the GSH and GSSG levels decreased and increased, respectively, at different stages of cirrhosis. There were no differences in the GSH/GSSG ratio and carbonylated protein content between groups. We also evaluated whether the active consumption of or abstinence from alcoholic beverages affected the behavior of these oxidative markers and only found differences in the MDA, GSH, and GSSG determination and the GSH/GSSG ratio. Our results suggest that alcoholic cirrhotic subjects have an increase in oxidative stress in the early stages of disease severity and that abstinence from alcohol consumption favors the major antioxidant endogen: GSH in patients with advanced disease severity.

## 1. Introduction

Alcohol is the most socially accepted addictive substance worldwide. The consumption of alcoholic beverages is a hallmark of social gatherings. However, in many societies, the consumption of these beverages in excess represents serious health and economic problems [1]. Chronic or excessive alcohol consumption can put physical and mental health at risk, damaging different organs such as the brain, liver, heart, lungs, skeletal musculature, and bones [2–4].

About 2–10% of absorbed alcohol is eliminated via the lungs and kidneys; the remainder is metabolized primarily by oxidative pathways in the liver and by nonoxidative pathways in the extrahepatic tissues. Oxidative metabolism in the liver is the result of extensive displacement of the liver's normal metabolic substrates, the production of acetaldehyde and reactive oxygen species (ROS), and an increase in the NADH/NAD^+^ ratio [5]. Data that demonstrate an increase in ROS production and a decrease in the antioxidant enzyme glutathione peroxidase-1 strongly suggest that chronic ethanol consumption creates an oxidative and potentially injurious environment within the hepatocyte, which could ultimately lead to oxidation and inactivation of cellular macromolecules. Lipid peroxidation [5] and oxidative alterations of mitochondrial DNA [6] have been observed after acute and chronic ethanol exposure. The pathogenic importance of the peroxidative process in ethanol-induced liver damage is still a subject of controversy. The positive evidence of enhanced lipid peroxidation in the liver has only been shown when animals are chronically fed with ethanol and given acute high doses of ethanol after overnight fasting or superimposed with a hypothermic condition [7]. In fact, only a few studies have examined the parameters of lipid peroxidation and hepatic content of antioxidants under a chronically intoxicated state.

Proteins are also an important target for oxidative damage because ROS can oxidize amino acid residues, cleave peptide bonds, increase protein fragmentation and aggregation, and alter proteolysis rates [5]. Thus, protein oxidation has to be considered one of several ethanol-related modifications that alter the functionality of proteins within the hepatocyte and especially within the mitochondrion, because ethanol increases ROS within this organelle. Protein modification elicited by the direct oxidative attack on the amino acid side chains by lipid peroxidation products, or as a consequence of reducing sugar, can lead to the generation of carbonyl groups within proteins [8]. On the other hand, reduced glutathione (GSH) is currently one of the most studied antioxidants. GSH is a natural compound made in the body from the amino acids glutamic acid, cysteine, and glycine. This molecule plays a crucial role in the body's detoxification process that occurs inside cells, mainly cells of the liver, kidney, intestines, and lungs. GSH has an especially important relationship with lipid peroxidation because of the known ability of such compounds to combine with free radicals that may initiate lipid peroxidation, as well as reduced hydrogen peroxide formed in cells [9]. Hepatic GSH has been observed to decrease after chronic alcohol consumption; this can be caused by acetaldehyde accumulation. Moreover, reduced GSH synthesis could also be a contributing factor to GSH depletion, as it has been documented in cirrhotic livers [10, 11]. The relationship between hepatic GSH and ethanol lipid peroxidation is unclear. GSH depression has been associated with ethanol-induced lipid peroxidation [11], but the depression may be either a result or a cause of the peroxidation.  Table 1 summarizes the oxidative markers produced by alcohol consumption quantified in this work.

The aim of the present work was to analyze whether oxidative stress had an important role in alcoholic liver disease. This was done by quantifying liver damage through the use of different oxidative markers. We found that oxidative molecules played an important role during the course of alcoholic liver disease; this role was more evident in the lipid damage and glutathione markers in patients with liver cirrhosis.

## 2. Materials and Methods

### 2.1. Patients

Alcohol dependence and abuse were assessed with the Diagnostic and Statistical Manual of Mental Disorders (DSM-IV) criteria and hazardous consumption was evaluated by the Alcohol Use Disorders Identification Test (AUDIT).

This cross-sectional study included 187 patients, eighteen years of age or older, who were divided into two groups: the control group (*n* = 130), which consisted of subjects with ethanol consumption ≤ 10 g/day and an AUDIT score ≤ 7, and the alcoholic liver cirrhosis (ALC) group (*n* = 57), which was made up of patients that presented with alcoholism in accordance with the World Health Organization (WHO) (ethanol consumption ≥ 70 g/day in men and ethanol consumption ≥ 50 g/day in women in the last 5 years) and a diagnosis of cirrhosis of the liver. The ALC group was then divided into 3 subgroups according to the patient Child-Pugh score: Child-Pugh A (*n* = 22), Child-Pugh B (*n* = 26), and Child-Pugh C (*n* = 9) [12].

Evaluation procedures included a detailed physical examination with anthropometry and assessment of the stigmata of nutritional deficiency.

Exclusion criteria for all groups were a positive viral panel, other concomitant liver damage, mental retardation, history of traumatic brain injury with loss of consciousness exceeding 10 min, and the presence of diseases that could affect the central nervous system.

The procedure was approved by the institutional review board. All participants provided written informed consent, and the study was carried out according to the provisions of the Declaration of Helsinki.

### 2.2. Chemicals

Trichloroacetic acid (TCA), hydrochloric acid, ethyl acetate, thiobarbituric acid, guanidine hydrochloride, and bovine albumin were purchased from Sigma Chemical Company (St. Louis, MO, USA). 2,4-Dinitrophenylhydrazine (DNPH), ethylic ethanol, and potassium dihydrogen phosphate were obtained from J.T. Baker (Xalostoc, Mexico).

### 2.3. Sample Collection

Blood samples (5 mL) were collected by venipuncture into Vacutainer tubes, with clot activators for serum collection and EDTA for blood and plasma collection for the biochemical assays and oxidative stress evaluation. Blood was immediately centrifuged for 10 minutes at 1308 ×g to collect the serum and the plasma.

Total blood was also utilized for glutathione determination.

### 2.4. Biochemical Analysis

Biochemical testing included blood analysis and liver function tests and was performed with automated systems (Vitros 250, Johnson & Johnson, New Jersey, USA, and Beckman Coulter HMX-AL Hematology Analyzer, California, USA). Results were negative for hepatitis B surface antigen (HBsAg) and hepatitis C virus (HCV) antibodies.

### 2.5. Reduced Glutathione, Oxidized Glutathione, and GSH/GSSG Ratio Determination in Whole Blood

We measured blood levels of reduced glutathione (GSH) and oxidized glutathione (GSSG) and the GSH/GSSG ratio by an enzymatic method using a commercially available kit (catalog number 371757, Calbiochem, Darmstadt, Germany) following the manufacturer's instructions. In short, the samples used to determine GSSG (100 *μ*L of whole blood mixed with 10 *μ*L of 1-methyl-2-vinylpiridinium trifluoromethane [scavenger]) and GSH (50 *μ*L of whole blood) were immediately frozen until their determination. Both samples were thawed and then mixed and incubated at room temperature for 2–10 minutes. The samples were acidified with 5% metaphosphoric acid and the supernatant was separated by centrifugation at 1000 ×g for 10 min at 4°C. For GSH and GSSG determination, we employed Ellman's reagent, which reacts with GSH to form a product detectable by spectrophotometry at 412 nm. GSSG could be determined by reducing GSSG to GSH, which was then determined by their action with Ellman's reagent. This method utilizes the change in color that occurs during the reaction, and the reaction rate is proportional to the GSH and GSSG concentrations. The calculation of the GSH and GSSG concentrations and the GSH/GSSG ratio requires four steps: (a) determination of the reaction rate, (b) calibration curves, (c) analyte concentration, and (d) calculation of the GSH/GSSG ratio. The GSH/GSSG ratio was calculated according to the following formula: (GSH-2GSSG)/GSSG [13]. The GSH/GSSG ratio decreases as a consequence of GSSG accumulation. Measurement of the GSSG level or determination of the GSH/GSSG ratio is a useful indicator of oxidative stress.

### 2.6. Carbonylated Protein Determination in Serum

DNPH was used for determining the carbonyl content in proteins. 200 *μ*L of 1 : 100 serum dilution was mixed with 200 *μ*L 20% TCA; the samples were mixed and then centrifuged (3290 ×g for 3 minutes). After centrifugation, the supernatant was decanted and 0.5 mL of 10 mM DNPH was added to the protein pellet. Sample blanks were prepared using 0.5 mL of 2.5 N HCl. The tubes were placed in a dark environment for one hour at room temperature and vortexed every 15 minutes; 0.5 mL of 20% TCA was added to each tube and then centrifuged (3 minutes at 3290 ×g). After centrifugation, the supernatant was decanted and 1 mL of ethanol-ethyl acetate solution was added. Following the mechanical disruption of the pellet by vortexing, the tubes were allowed to stand for 10 minutes and then spun again (3 minutes at 3290 ×g). The supernatant was decanted and the pellet washed with ethanol-ethyl acetate two more times. After the final wash, the protein was solubilized in 1 mL of 6 M guanidine hydrochloride and 20 mM potassium dihydrogen phosphate (pH 2.3). To speed up the solubilization process, the samples were incubated in a 37°C water bath for 15 minutes. The final solution was centrifuged to remove any insoluble material. The carbonyl content was calculated from the absorbance measurement at 360, 370, and 390 nm and an absorption coefficient = 22000 M^−1^ cm^−1^ [14].

### 2.7. Lipid Peroxidation Assessment

Lipid peroxidation was estimated in the serum samples by measuring the malondialdehyde (MDA) formation using the thiobarbituric acid method [15]. Briefly, 100 *μ*L of serum of alcoholic patients or control subjects was mixed with 500 *μ*L of 150 mM Tris-HCl and 1.5 mL of 0.375% TBA and vortexed for 10 seconds. The reaction mixture was then incubated at 100°C for 45 minutes in a water bath. At the end of incubation, the samples were centrifuged at 1000 ×g for 10 minutes. The MDA content was calculated from the absorbance measurement at 532 nm and an absorption coefficient = 1.56 × 10^5^ cm^−1^ M^−1^.

Total protein was determined according to Bradford [16], using bovine serum albumin as the standard.

### 2.8. Statistical Analysis

Data were expressed as mean values ± SEM. Comparisons were carried out by analysis of variance (ANOVA) and orthogonal contrasts were used to determine the differences between all groups. Correlations were calculated with Spearman's rank correlation, as required. The analyses were carried out with the Windows SPSS 15.0 statistical software (SPSS Inc., Chicago, IL). Differences were considered statistically significant when the *p* value was less than 0.05.

## 3. Results

Table 2 shows the main demographic characteristics of our study population. We included 130 participants in the control group and 57 patients with alcoholic liver cirrhosis that were classified into subgroups, according to the Child-Pugh score, as Child-Pugh A, Child-Pugh B, or Child-Pugh C. Men predominated in all the study groups. The grams of ethanol consumed in a day for the patients with ALC were higher than those of the control group (*p* ≤ 0.05). There was also a significant difference in relation to age between the Child-Pugh subgroups of the ALC patients; older patients had more severe disease (*p* < 0.05). Body mass index values are also shown in Table 2.

Serum markers of liver damage (AST, GGT, and albumin) were significantly altered according to liver damage progression (Table 3). The AST and GGT enzyme levels were higher in the ALC patients, compared with the control group, and the results were statistically significant (*p* ≤ 0.05). Differences were also observed between the Child-Pugh A versus Child-Pugh B subgroups and the Child-Pugh A versus Child-Pugh C subgroups in regard to AST levels (*p* ≤ 0.05). Table 3 also shows the levels of hemoglobin, hematocrit, and platelets. A directly proportional decrease in the levels of each marker was observed in all the cases with respect to the severity of damage and these results were statistically significant.

**Figure 1:**
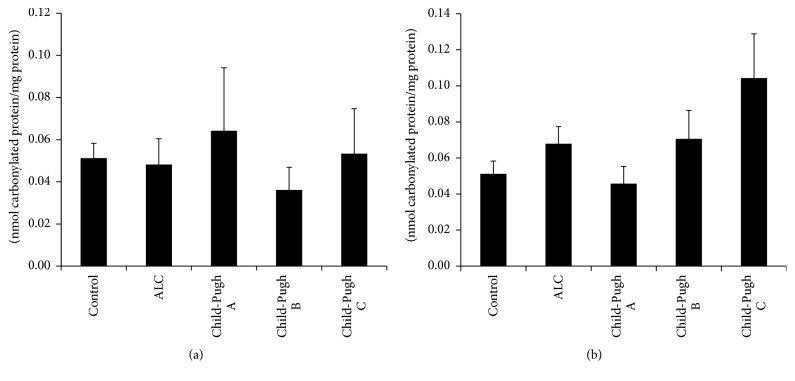
Carbonylated protein determination. Oxidative damage determined as carbonylated protein levels in serum from participants of the control group (control) and patients with alcoholic liver cirrhosis (ALC) classified according to the Child-Pugh score as Child-Pugh A, Child-Pugh B, or Child-Pugh C. (a) Patients without current alcohol consumption; (b) patients with active alcohol consumption. Each bar represents the mean value ± SEM. Differences were considered statistically significant when the *p* value was less than 0.05.

**Figure 2:**
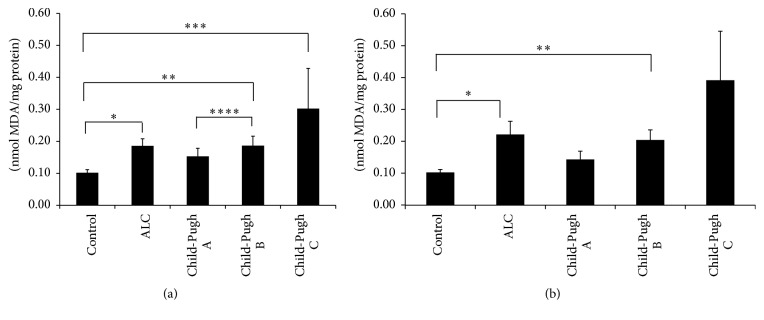
Evaluation of oxidative damage in lipids. Lipid peroxidation determined as malondialdehyde (MDA) content in serum samples from participants of the control group (control) and patients with alcoholic liver cirrhosis (ALC), classified according to the Child-Pugh score as Child-Pugh A, Child-Pugh B, or Child-Pugh C. (a) Patients without current alcohol consumption; (b) patients with active alcohol consumption. Each bar represents the mean value ± SEM. For (a): ^*∗*^ *p* = 0.001, control group versus ALC group; ^*∗∗*^ *p* = 0.004, control group versus Child-Pugh B subgroup; ^*∗∗∗*^ *p* = 0.003, control group versus Child-Pugh C subgroup; ^*∗∗∗∗*^ *p* = 0.049, Child-Pugh A versus Child-Pugh B subgroup. For (b): ^*∗*^ *p* = 0.036, control group versus ALC group; ^*∗∗*^ *p* = 0.013, control group versus Child-Pugh B subgroup.

Various studies* in vitro* and* in vivo* suggest that oxidative stress plays an important role in the development of ALC [17].  Table 4 shows the results of several oxidative damage markers. The differences between MDA, GSH, and GSSG levels of the control group and the patients with cirrhosis of the liver were statistically significant. The MDA levels increased in proportion to the severity of damage, whereas the GSH and GSSG levels decreased and increased, respectively, in the Child-Pugh A subgroup but recovered at other stages of cirrhosis (Child-Pugh B and Child-Pugh C). There were no differences in the GSH/GSSG ratio and carbonylated protein content between groups. In addition, to evaluate whether active consumption of or abstinence from alcoholic beverages by the patients affected the behavior of these oxidative markers, we classified the patients according to the following criteria: 11 patients (Child-Pugh A), 9 patients (Child-Pugh B), and 6 patients (Child-Pugh C) were active consumers in the last year, and 11 patients (Child-Pugh A), 17 patients (Child-Pugh B), and 3 patients (Child-Pugh C) were abstainers (patients with no alcohol consumption during the last 6 months). Similar to results shown in Table 4, there were no significant differences between study groups in relation to carbonylated protein content (Figures 1(a) and 1(b)). On the other hand, as shown in Figure 2, the MDA levels increased in the nondrinking ALC patients and also in accordance with the Child-Pugh score (*p* ≤ 0.05). With respect to the control group (Figure 2(a)), MDA concentration was higher according to liver disease severity and the pattern was the same in the ALC patients with active alcohol consumption (Figure 2(b)).

**Figure 3:**
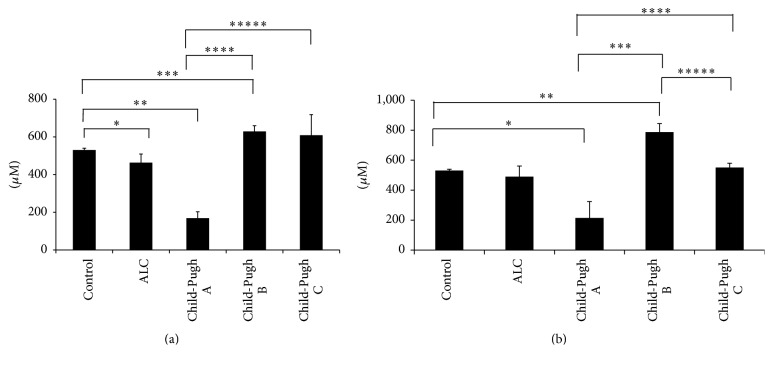
GSH level determination. Reduced glutathione (GSH) content was determined in total blood from participants of the control group (control) and patients with alcoholic liver cirrhosis (ALC), classified according to the Child-Pugh score as Child-Pugh A, Child-Pugh B, or Child-Pugh C. (a) Patients without current alcohol consumption; (b) patients with active alcohol consumption. Each bar represents the mean value ± SEM. For (a): ^*∗*^ *p* = 0.022, control group versus ALC group; ^*∗∗*^ *p* = 0.001, control group versus Child-Pugh A subgroup; ^*∗∗∗*^ *p* = 0.001, control group versus Child-Pugh B patients; ^*∗∗∗∗*^ *p* = 0.001, Child-Pugh A versus Child-Pugh B subgroups; ^*∗∗∗∗∗*^ *p* = 0.001, Child-Pugh A subgroup versus Child-Pugh C patients. For (b): ^*∗*^ *p* = 0.016, control group versus Child-Pugh A subgroup; ^*∗∗*^ *p* = 0.02, control group versus Child-Pugh B patients; ^*∗∗∗*^ *p* = 0.001, Child-Pugh A versus Child-Pugh B subgroups; ^*∗∗∗∗*^ *p* = 0.012, Child-Pugh A subgroup versus Child-Pugh C patients; ^*∗∗∗∗∗*^ *p* = 0.003, Child-Pugh B patients versus Child-Pugh C subgroup.

**Figure 4 fig4:**
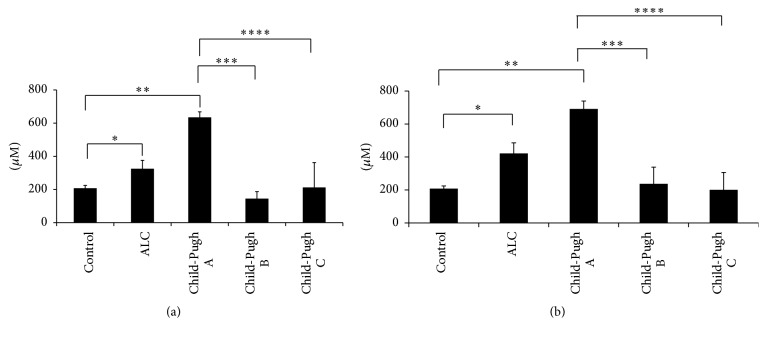
GSSG level determination. Oxidized glutathione (GSSG) levels were determined in total blood from participants of the control group (control) and patients with alcoholic liver cirrhosis (ALC), classified according to the Child-Pugh score as Child-Pugh A, Child-Pugh B, or Child-Pugh C. (a) Patients without current alcohol consumption; (b) patients with active alcohol consumption. Each bar represents the mean value ± SEM. For (a): ^*∗*^ *p* = 0.008, control group versus ALC group; ^*∗∗*^ *p* = 0.001, control group versus Child-Pugh A subgroup; ^*∗∗∗*^ *p* = 0.001, Child-Pugh A versus Child-Pugh B subgroups; ^*∗∗∗∗*^ *p* = 0.001, Child-Pugh A subgroup versus Child-Pugh C patients. For (b): ^*∗*^ *p* = 0.001, control group versus ALC group; ^*∗∗*^ *p* = 0.001, control group versus Child-Pugh A subgroup; ^*∗∗∗*^ *p* = 0.001, Child-Pugh A versus Child-Pugh B subgroups; ^*∗∗∗∗*^ *p* = 0.001, Child-Pugh A subgroup versus Child-Pugh C patients.

**Figure 5:**
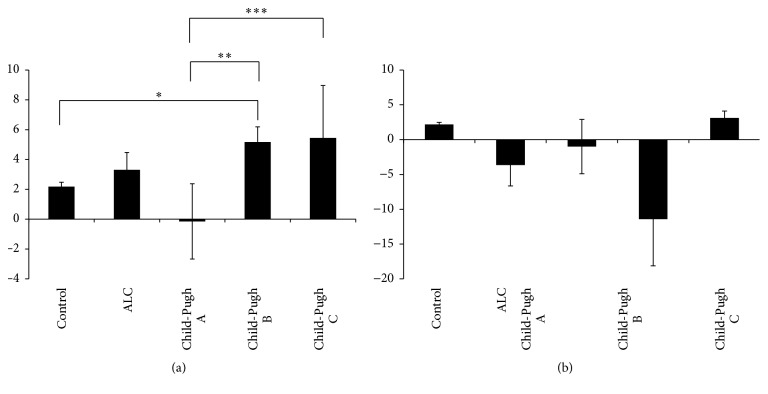
GSH/GSSG ratio determination. The GSH/GSSG ratio was determined in total blood from participants of the control group (control) and patients with alcoholic liver cirrhosis (ALC), classified according to the Child-Pugh score as Child-Pugh A, Child-Pugh B, or Child-Pugh C. (a) Patients without current alcohol consumption; (b) patients with active alcohol consumption. Each bar represents the mean value of experiments performed ± SEM. For (a): ^*∗*^ *p* = 0.004, control group versus Child-Pugh B patients; ^*∗∗*^ *p* = 0.001, Child-Pugh A versus Child-Pugh B subgroups; ^*∗∗∗*^ *p* = 0.034, Child-Pugh A subgroup versus Child-Pugh C patients.

GSH behavior in all cirrhotic patients was different, regardless of active alcohol consumption or abstinence. In the Child-Pugh A subgroup, GSH levels decreased significantly in the abstainers, as well as in the active consumers, compared with the control group. The levels of this tripeptide recovered in the Child-Pugh B patients with and without alcohol consumption, but they decreased once more in patients with active alcohol consumption (Child-Pugh C subgroup) (Figures 3(a) and 3(b)). Conversely, Figures 4(a) and 4(b) show that GSSG increased during the development of liver damage, being the highest in patients classified with Child-Pugh A, with respect to the control group, as well as to the Child-Pugh B and Child-Pugh C patients. This response was similar for both abstinence from alcohol and its active consumption (Figures 4(a) and 4(b)). Finally, the GSH/GSSG ratio had a positive direction in the abstinent patients (*p* ≤ 0.05), showing that they had a higher concentration of reduced glutathione (Figure 5(a)). This response was the opposite in the active consumers (Figure 5(b)), indicating that there was a tendency to have a higher concentration of oxidized glutathione. Therefore, active alcohol consumption had a tendency to produce glutathione oxidation, whereas abstinence from alcohol consumption restored the main antioxidant molecule, reduced glutathione.

We found that the Child-Pugh Score was directly related to AST (*r*_*s*_ = 0.522, *p* < 0.001), albumin (*r*_*s*_ = 0.707, *p* < 0.001), MDA (*r*_*s*_ = 0.395, *p* = 0.002), GSH (*r*_*s*_ = 0.589, *p* < 0.001), and GSSG (*r*_*s*_ = −0.657, *p* < 0.001). The GSH levels were related to albumin (*r*_*s*_ = −0.484, *p* < 0.001) and the GSSG (*r*_*s*_ = −0.546, *p* < 0.001) and MDA levels with VCM (*r*_*s*_ = −0.458, *p* < 0.001) and GSSG (*r*_*s*_ = 0.277, *p* = 0.037).

## 4. Discussion

ALD is a common response to and consequence of long-term ethanol abuse and represents a major cause of morbidity and mortality worldwide. The pathophysiology of this damage involves different stages, including steatosis, steatohepatitis, fibrosis, and cirrhosis, and a small percentage of patients with established cirrhosis develop hepatocellular carcinoma [18]. The mechanisms involved in ALC pathogenesis are immune and inflammatory responses, genetic factors, and the oxidative stress generated during hepatic alcohol metabolism.

Different studies have reported on oxidative stress participation in ALC. In 1963, Di Luzio [19], followed by other studies, showed that ethanol promotes the formation of a variety of free radical intermediates by several cell types, including hepatocytes, Kupffer cells, endothelial cells, and infiltrating inflammatory leucocytes [20, 21]. Several studies have also demonstrated that supplementation with different antioxidants and free radical scavengers reduced hepatic injury in alcohol-fed rodents [22–24]. In the present work, we studied the behavior of several oxidative stress markers in serum and blood samples of patients with ALC, in specific liver cirrhosis that was also classified according to the Child-Pugh score.

Different reports indicate that oxidative stress, specifically oxidative mitochondrial damage, can be responsible for hepatocyte apoptosis/necrosis in ALC [25]. This explains the results obtained in the ALT, AST, and GGT levels. The oxidative stress induction within liver mitochondria is associated with the collapse of the mitochondrial membrane potential and the onset of mitochondrial permeability transition (MPT) [26]. MPT is characterized by the opening of a megachannel in the mitochondrial membrane as a result of protein complex assembly [27]. Extensive MPT leads to mitochondrial swelling due to the influx of ions and water and is critical for the onset of hepatocyte death by necrosis [28]. Such a response explains the increase in liver damage markers we found in our patients. GGT is an enzyme derived from the plasma membrane of hepatocytes and its activity has been accepted as a biomarker of ALC [29]. This enzyme is involved in the transfer of *γ*-glutamyl peptides to amino acids and in the synthesis of GSH, hydrolyzing GSH to its amino acid components. The enzyme cysteine is used for intracellular resynthesis [30, 31] and thus plays an important role in the antioxidant defense system.

Our results showed that GGT activity increased and GSH levels decreased in the ALC patients. The GSH reduction in the Child-Pugh A patients was more evident than in the Child-Pugh B or Child-Pugh C patients. This decrease in the GSH levels could be explained by the years of alcohol consumption, according to Child-Pugh A, Child-Pugh B, and Child-Pugh C, which were 28, 26, and 22 years, respectively (data not shown).

Alcohol consumption may contribute to secondary anemia due to its direct effects on the liver and also to other different mechanisms [32]. Folic acid and vitamin B12 deficiencies frequently develop in patients with cirrhosis. These changes may be related to inadequate food intake or intestinal malabsorption. Folic acid deficiency is the most common cause of low hematocrit in alcoholic patients [33, 34]. Anemia in an alcoholic person may also be a consequence of the direct toxic effects on the erythrocyte precursor in bone marrow [35]. These observations may explain our study results in which the levels of hemoglobin and hematocrit decreased in relation to the severity of damage.

Ethanol consumption leads to the generation of ROS, which can potentially damage any biological molecules (proteins, lipids, or DNA). However, proteins are possibly the most immediate vehicle for inflicting oxidative damage on cells because they are often catalysts, rather than stoichiometric mediators. Hence, the effect of damage to one molecule is greater than a stoichiometric change [36]. Protein carbonyl content is actually the most general indicator and by far the most commonly used marker of protein oxidation [17, 37]. Carbonyl groups (aldehydes and ketones) are produced on protein side chains (especially of Pro, Arg, Lys, and Thr) when they are oxidized. These moieties are chemically stable, which is useful for both their detection and storage [36]. In our study, carbonylated protein levels did not show statistically significant differences between the control group and the patients with ALC (Child-Pugh A, Child-Pugh B, or Child-Pugh C), even taking into account the current consumption or abstinence of our participants.

We also evaluated lipid peroxidation through malondialdehyde quantification. Polyunsaturated lipids are essential to the entire support system of the cell, including cell membranes, the endoplasmic reticulum, and the mitochondria. Disruption of their structural properties can therefore have dire consequences on cellular function. Peroxidation of lipids has been thought to be a major effect of free radicals. Because of this, many of the assay methods for establishing free radical-induced injury have measured products of the reaction of these molecules with lipids; one of these products is malondialdehyde [38, 39]. The pathogenic importance of this peroxidative process in ethanol-induced liver injury is subject to debate. In fact, only a few studies have examined the parameters of lipid peroxidation and hepatic content of antioxidants under a chronically intoxicated state. Drinking more than two standard drinks per day over a long period of time may be associated with significant elevation of iron overload in tissue [40]. In animal models, iron and alcohol have been shown to act in a synergistic manner to enhance lipid peroxidation, leading to the formation of MDA [10]. Furthermore, it is known that patients with ALC have antibodies targeting cytochrome P450 2E1 and oxidized phospholipids. Preclinical and clinical studies show that the elevation of IgG against lipid peroxidation-derived antigens is associated with TNF-*α* elevation and the severity of liver inflammation [41]. Our results demonstrated that the serum levels of MDA were significantly higher in patients with cirrhosis of the liver and in the same order in subjects with different Child-Pugh scores. In regard to both active alcohol consumption and abstinence from alcohol, peroxidative damage was maintained.

There is much information about the effect of ethanol consumption and antioxidant defense depletion. GSH is the most important nonenzymatic antioxidant present in cells. Early studies have shown that a decrease in GSH levels and an increase in GSSG levels in the liver, regardless of nutritional status or the extent of liver disease, are a common feature in ethanol-fed animals, as well as in patients with alcoholism [19]. GSH homeostasis is very important in the prevention of alcohol-mediated oxidative injury. This statement is supported by the observation that the stimulation of GSH resynthesis in rats by supplementation with GSH precursors, such as N-acetylcysteine, prevents liver damage in the enteral alcohol model [30]. In our study, we observed the progressive changes in the levels of GSH, GSSG, and the GSH/GSSG ratio. In the first phases of liver damage, all parameters were altered, the most evident of which was the oxidative stress (the concentration of oxidized glutathione was higher than reduced glutathione as an outcome of GSSG accumulation) in patients with Child-Pugh A. It is known that short periods of alcohol consumption stimulate GSH depletion [11, 12], but Tietze in 1969 observed that, in long-term alcohol feeding in rats, the GSH levels increased. The mechanism of this response is unclear, but evidence suggests that depletion or increased turnover of glutathione due to ethanol is caused by the formation of acetaldehyde adducts [21], impaired glutathione synthesis [42], or increased losses from the liver tissue [43]. Our results of patients with ALC according to the Child-Pugh score were similar to those obtained in preclinical studies. Perhaps this was a compensatory response to counteract the alcohol damage in long-term alcohol consumption (drinkers for at least 22 years).

Null alcohol consumption modified the behavior of GSH and GSSG, in particular in Child-Pugh B and Child-Pugh C patients. They showed increased GSH levels and lower GSSG concentration when compared with the Child-Pugh A patients. In addition, the GSH/GSSG ratio values were positive in the Child-Pugh B and Child-Pugh C groups, as a consequence of GSH accumulation, which favors antioxidation (Figures 3 [Fig fig4]–5) and possibly stops different inflammatory mediators, such as cytokines. However, the GHS/GSSG ratio in patients with active consumption showed a negative trend, which is manifested as the presence of oxidative liver damage. These results must be confirmed in a larger number of patients in each Child-Pugh group. The number of patients in the present study was a limitation.

The free radical-antioxidant imbalance is possibly one of the major factors that contributes to the prevalence of mortality in the Mexican population, even though alcohol consumption* per capita *is moderate, compared with that of European countries [44].

Oxidative stress markers have normally been measured in liver tissue. Our results indicate that it is possible to measure them in peripheral blood, reflecting what is occurring in the liver in patients with alcohol liver cirrhosis.

Given our findings, we consider it necessary to carry out clinical trials that evaluate the use of antioxidants and the use of antioxidant therapy as a possible support therapy plus treatment to counteract liver damage induced by alcohol consumption.

## 5. Conclusions

Our results are the first to be reported on oxidative markers in a Latin American population. They suggest that alcoholic cirrhotic subjects have an increase in oxidative stress in the early stages of disease severity and that abstinence from alcohol consumption favors the major antioxidant endogen: GSH in patients with advanced disease severity.

## Figures and Tables

**Table 1:** Biochemical markers of liver damage.

Liver damage markers	Level of damage
(i) MDA levels 4-Hydroxy-2,3-nonenal 4-Hydroxy-2,3-alkenal	(i) Damage in cellular membranes or lipid level
(ii) Carbonylated protein levels	(ii) Damage in biologically active proteins
(iii) GSH and GSSG quantification	(iii) Cytoplasmic damage

MDA: malondialdehyde; GSH: reduced glutathione; GSSG: oxidized glutathione.

**Table 2:** Results of clinical parameters of the subjects included in the study.

	Control	Patients with ALC			
		ALC	Child-Pugh A	Child-Pugh B	Child-Pugh C
Sex, F/M (%)	39/91 (30/70)	2/55 (3.5/96.4)	1/21 (1.8/36.8)	1/25 (1.8/43.8)	0/9 (0/15.7)
Age (years)	38.0 ± 1.4	49.3 ± 1.2^*∗*^	45.9 ± 2.7^*∗∗*^	47.2 ± 1.9^*∗∗∗*^	53.2 ± 3.5^*∗∗∗∗∗∗*^
Body mass index (kg/m^2^)	28.3 ± 0.3	28.8 ± 0.6	29.5 ± 1.1	27.9 ± 0.8	29.9 ± 1.9
Consumption (g OH/day)	0.9 ± 0.1	304.1 ± 29.5^*∗*^	372.3 ± 66.6^*∗∗*^	257.9 ± 25.9^*∗∗∗*^	271.1 ± 41.3^*∗∗∗∗*^

Values represented as the mean ± SEM. ^*∗*^ *p* ≤ 0.05, control group versus ALC group; ^*∗∗*^ *p* ≤ 0.05, control group versus Child-Pugh A subgroup; ^*∗∗∗*^ *p* ≤ 0.05, control group versus Child-Pugh B patients; ^*∗∗∗∗*^ *p* ≤ 0.05, control group versus Child-Pugh C subgroup; ^*∗∗∗∗∗∗*^ *p* ≤ 0.05, Child-Pugh A subgroup versus Child-Pugh C patients.

**Table 3:** Results of biochemical parameters evaluated in serum and blood of the study participants.

	Control	Patients with ALC			
		ALC	Child-Pugh A	Child-Pugh B	Child-Pugh C
AST (UI/L)	29.9 ± 0.9	50.2 ± 3.3^*∗*^	37.7 ± 2.5^*∗∗*^	50.5 ± 3.5^*∗∗∗*,*∗∗∗∗∗*^	78.5 ± 14.01^*∗∗∗∗*,*∗∗∗∗∗∗*^
ALT (UI/L)	27.8 ± 1.6	33.1 ± 2.3	31.4 ± 3.2	34.4 ± 4.03	33.7 ± 4.1
GGT (UI/L)	31.5 ± 2.4	110.7 ± 13.1^*∗*^	79.3 ± 13.4^*∗∗*^	126.9 ± 20.8^*∗∗∗*^	133.8 ± 44.1^*∗∗∗∗*^
Albumin (g/dL)	4.4 ± 0.03	3.2 ± 0.1^*∗*^	3.9 ± 0.08^*∗∗*^	2.9 ± 0.1^*∗∗∗*,*∗∗∗∗∗*^	2.3 ± 0.2^*∗∗∗∗*,*∗∗∗∗∗∗*,*∗∗∗∗∗∗∗*^
Hb (g/dL)	16.4 ± 0.1	12.8 ± 0.4^*∗*^	13.8 ± 0.6^*∗∗*^	12.4 ± 0.5^*∗∗∗*^	11.8 ± 1.1^*∗∗∗∗*^
Hct (%)	49.6 ± 0.3	38.5 ± 1.1^*∗*^	41.9 ± 1.6^*∗∗*^	36.9 ± 1.6^*∗∗∗*,*∗∗∗∗∗*^	35 ± 3.01^*∗∗∗∗*^
Platelets (mm^3^)	270.5 ± 4.9	139.5 ± 12.3^*∗*^	144.3 ± 20.08^*∗∗*^	147.2 ± 20.7^*∗∗∗*^	105.8 ± 11.4^*∗∗∗∗*^

AST: aspartate aminotransferase; ALT: alanine aminotransferase; GGT: gamma-glutamyl transpeptidase; Hb: hemoglobin; Hct: hematocrit. Values represented as the mean ± SEM. ^*∗*^ *p* ≤ 0.05, control group versus ALC group; ^*∗∗*^ *p* ≤ 0.05, control group versus Child-Pugh A subgroup; ^*∗∗∗*^ *p* ≤ 0.05, control group versus Child-Pugh B patients; ^*∗∗∗∗*^ *p* ≤ 0.05, control group versus Child-Pugh C subgroup; ^*∗∗∗∗∗*^ *p* ≤ 0.05, Child-Pugh A versus Child-Pugh B subgroups; ^*∗∗∗∗∗∗*^ *p* ≤ 0.05, Child-Pugh A subgroup versus Child-Pugh C patients; ^*∗∗∗∗∗∗∗*^ *p* ≤ 0.05, Child-Pugh B patients versus Child-Pugh C subgroup.

**Table 4:** Results of oxidative stress markers determined in serum and total blood of the study participants.

	Control	Patients with ALC			
		ALC	Child-Pugh A	Child-Pugh B	Child-Pugh C
Sex, F/M (%)	39/91 (30/70)	2/55 (3.5/96.4)	1/21 (1.8/36.8)	1/25 (1.8/43.8)	0/9 (0/15.7)
Carbonylated proteins (nmol carbonylated prot./mg prot.)	0.05 ± 0.007	0.05 ± 0.007	0.05 ± 0.01	0.04 ± 0.01	0.08 ± 0.01
MDA (nmol MDA/mg prot.)	0.1 ± 0.01	0.2 ± 0.02^*∗*^	0.18 ± 0.02^*∗∗*^	0.2 ± 0.02^*∗∗∗*^	0.3 ± 0.1^*∗∗∗∗*^
GSH (*μ*M)	530.6 ± 9.1	475.5 ± 40.3	191. 3 ± 56.3^*∗∗*^	683.1 ± 31.3^*∗∗∗*,*∗∗∗∗∗*^	570.3 ± 37.7^*∗∗∗∗∗∗*,*∗∗∗∗∗∗∗*^
GSSG (*μ*M)	207.8 ± 16.6	368.9 ± 40.4^*∗*^	663.3 ± 29.01^*∗∗*^	176.8 ± 44.1^*∗∗∗∗∗*^	204.6 ± 80.8^*∗∗∗∗∗∗*^
GSH/GSSG ratio	2.1 ± 0.2	0.1 ± 1.5	−0.5 ± 2.2	−0.5 ± 2.8	3.8 ± 1.2

Values represented as the mean ± SEM. ^*∗*^ *p* ≤ 0.05, control group versus ALC group; ^*∗∗*^ *p* ≤ 0.05, control group versus Child-Pugh A subgroup; ^*∗∗∗*^ *p* ≤ 0.05, control group versus Child-Pugh B patients; ^*∗∗∗∗*^ *p* ≤ 0.05, control group versus Child-Pugh C subgroup; ^*∗∗∗∗∗*^ *p* ≤ 0.05, Child-Pugh A versus Child-Pugh B subgroups; ^*∗∗∗∗∗∗*^ *p* ≤ 0.05, Child-Pugh A subgroup versus Child-Pugh C patients; ^*∗∗∗∗∗∗∗*^ *p* ≤ 0.05, Child-Pugh B patients versus Child-Pugh C subgroup.
